# Supplementary material for: Splice-Junction-Based Mapping of Alternative Isoforms in the Human Proteome
Source: Cell Rep. Author manuscript; Available in PMC 2020 Jan 15. (PMC6961840; doi:10.1016/j.celrep.2019.11.026)
Supplement: 2 [file NIHMS1546469-supplement-2.zip › DF1/PXD009737/TestisTrypsin/TestisTrypsin_59_CLUH_QPQGLGCLPFLPERAGQSAAGSCSLK.pdf]

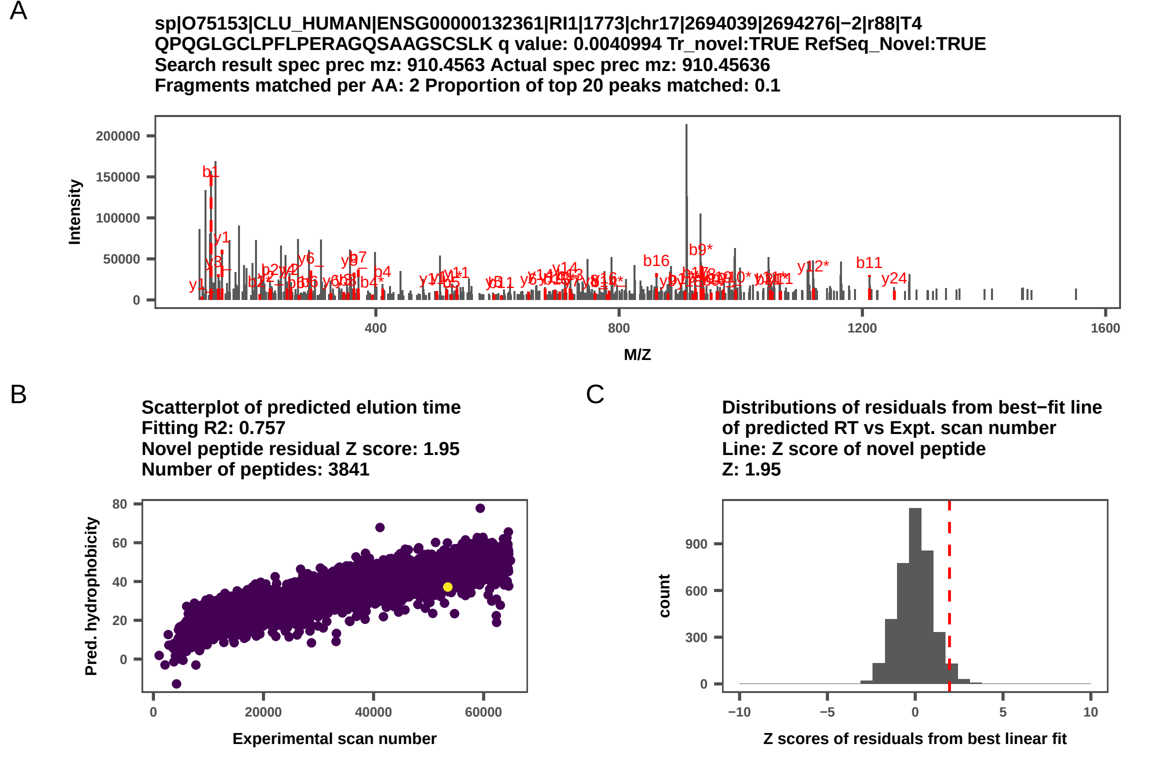

A
sp|O75153|CLU_HUMAN|ENSG00000132361|RI1|1773|chr17|2694039|2694276|−2|r88|T4
QPQGLGCLPFLPERAGQSAAGSCSLK q value: 0.0040994 Tr_novel:TRUE RefSeq_Novel:TRUE
Search result spec prec mz: 910.4563 Actual spec prec mz: 910.45636
Fragments matched per AA: 2 Proportion of top 20 peaks matched: 0.1
Intensity
200000
150000
100000
50000
0
400
800
1200
1600
M/Z
b1
y1
b16
b9*
b11
y12*
y24
B
Scatterplot of predicted elution time
Fitting R2: 0.757
Novel peptide residual Z score: 1.95
Number of peptides: 3841
Pred. hydrophobicity
80
60
40
20
0
0
20000
40000
60000
Experimental scan number
C
Distributions of residuals from best−fit line
of predicted RT vs Expt. scan number
Line: Z score of novel peptide
Z: 1.95
count
900
600
300
0
−10
−5
0
5
10
Z scores of residuals from best linear fit
